# Developing a clinical teaching quality questionnaire for use in a university osteopathic pre-registration teaching program

**DOI:** 10.1186/s12909-015-0358-6

**Published:** 2015-04-08

**Authors:** Brett Vaughan

**Affiliations:** 1 Centre for Chronic Disease Prevention & Management, College of Health & Biomedicine, Victoria University, Melbourne, Australia; 2 Institute of Sport, Exercise & Active Living, Victoria University, Melbourne, Australia; 3 School of Health & Human Sciences, Southern Cross University, Lismore, Australia

**Keywords:** Evaluation, Exploratory factor analysis, Student-led clinic, Student-run clinic, Clinical education, Osteopathy, Osteopathic medicine

## Abstract

**Background:**

Clinical education is an important component of many health professional training programs. There is a range of questionnaires to assess the quality of the clinical educator however none are in student-led clinic environments. The present study developed a questionnaire to assess the quality of the clinical educators in the osteopathy program at Victoria University.

**Methods:**

A systematic search of the literature was used to identify questionnaires that evaluated the quality of clinical teaching. Eighty-three items were extracted and reviewed for their appropriateness to include in a questionnaire by students, clinical educators and academics. A fifty-six item questionnaire was then trialled with osteopathy students. A variety of statistics were used to determine the number of factors to extract. Exploratory factor analysis (EFA) was used to investigate the factor structure.

**Results:**

The number of factors to extract was calculated to be between 3 and 6. Review of the factor structures suggested the most appropriate fit was four and five factors. The EFA of the four-factor solution collapsed into three factors. The five-factor solution demonstrated the most stable structure. Internal consistency of the five-factor solution was greater than 0.70.

**Conclusions:**

The five factors were labelled Learning Environment (Factor 1), Reflective Practice (Factor 2), Feedback (Factor 3) and Patient Management (Factor 4) and Modelling (Factor 5). Further research is now required to continue investigating the construct validity and reliability of the questionnaire.

**Electronic supplementary material:**

The online version of this article (doi:10.1186/s12909-015-0358-6) contains supplementary material, which is available to authorized users.

## Background

Clinical education is an important component of health profession education programs, as it provides an opportunity for students to apply the skills and knowledge they have learnt in the classroom in an ‘authentic’ learning environment [1-3]. Clinical education usually takes the form of student management of patients under the supervision of related qualified health professionals with placement type influencing the volume and type of teaching and/or supervision [4], the type of health care provided, and degree of student involvement in health care events.

Authors have described the educational theories that may be applied to clinical education and these typically focus on those that related to workplace learning [5-7]. Although there has been no explicit discussion of the theories underlying osteopathic clinical education, Vaughan et al. [8] suggest that the Cognitive Apprenticeship model could account for aspects of the learning and student-educator interaction that takes place in the on-campus, student-led clinics. Beyond the commentary by these authors, we must explore the wider health profession education literature in order to draw on other theories. The profession with the most similarities from an education and professional practice viewpoint is physiotherapy. Patton et al. [5] highlight there has been little in the way of literature published on the theories that underpin clinical education in physiotherapy. The subsequent commentary by these authors suggests, “…that one model or specification could address the needs of every situation would be contestable.” These authors describe workplace learning, learning as practice, social learning, situated learning and reflective/critical thinking as models that can be applied to clinical education in physiotherapy. It is likely that these models are also applicable to osteopathic clinical education and readers are encouraged to review the work by Patton et al. [5] for a comprehensive description of these models.

Teaching in a clinical environment is complex [3,9,10]. It includes issues related to patients such as safety and patient census - the availability and variety of patients and illnesses, clinic operational issues such as timetables and facilities, issues related to students such as time management [3], and individual characteristics issues related to the clinical educator such as personality [4,11] and education. Cross [12] reported that students perceived there was a strong relationship between being a good physiotherapist and a good clinical teacher, however this does not appear to be a consistent theme that emerges from the literature nor is there strong evidence that this relationship improves student learning.

We know that clinical educators require clinical competency [2,13]; good clinical reasoning skills [2,12]; appropriate, relevant and up-to-date knowledge [14]; good interpersonal skills; and supervision and teaching skills [12,14-18]. These attributes also extend to the provision of timely student feedback [14,18-23], regular observation of students [10], role-modelling [10,12,17,24-29] and the development of a positive, professional and supportive learning environment [15,22,30,31]. Sutkin et al. [15] provide an extensive list of characteristics of a ‘good’ clinical educator based on their systematic search of the literature.

Although the list of clinical educator attributes is extensive, there is no research that consistently demonstrates which attributes contribute to effective student learning [32] and further research is required [14]. Students have an opinion and expectation as to what constitutes a good clinical educator [10]. That said, arguably, one of the most effective ways of determining the impact of clinical educator attributes on students learning is to explore students perspectives [14].

Assessing the teaching quality is one part of a course evaluation strategy used to help inform the quality cycle necessary for review, improvement and program accreditation. Student ratings are already widely used to explore the quality of clinical teaching [33,34]. For that reason, there are a large and growing number of clinical teaching quality questionnaires in the literature with systematic reviews of available questionnaires by Fluit et al. [35] and Beckman et al. [33]. As with any performance measure, the validity, reliability and feasibility of a questionnaire are important to investigate and establish [14,36], particularly where the results of the questionnaire are used for employment decisions or performance appraisals. Ideally questionnaires should be convenient for the student to complete with the results providing motivation for clinical educators to continue to improve their teaching [22].

Clinical teaching in osteopathic education outside of the United States typically takes place in out-patient or on-campus clinics where students manage and treat patients under the supervision of qualified osteopaths (the osteopathic clinical educator). Senior students in the osteopathy program take on the responsibility of conducting the entire patient consultation including taking a clinical/medical history, physical examination, manual therapy treatment, and provision of advice related to exercise and lifestyle factors as part of the management of the patients’ presenting complaint. Supervision of the student is provided by qualified osteopaths in a ratio of 1 educator for every 5–6 students. This ratio is different to other professions such as physiotherapy [37,38] and occupational therapy [39] where 1:1 or 1:2 ratios are common. The role of the osteopathic clinical educator is fourfold: 1) to ensure that the student is performing a safe and effective consultation; 2) support the student through the experience of managing patients with a variety of musculoskeletal and concomitant psychosocial issues; 3) encourage the student to reflect on their patient management; and 4) propose alternative patient management strategies. These roles are consistent with idea of ‘supported participation’ as a model for learning in a clinical environment as described by Dornan et al. [6]. The role may also occasionally require the clinical educator to perform aspects of the examination or treatment, and this provides a limited opportunity to role model patient management skills. Literature regarding osteopathic clinical education and clinical educators in Australia, New Zealand and the United Kingdom is beginning to emerge [8]. However, research into osteopathic clinical education is required. Further, there is a clear need to investigate the students’ perception of the quality of clinical teaching in an osteopathic student-led teaching clinic. The current paper reports on the development of a questionnaire to assess clinical teaching in osteopathic clinical education in on-campus, student-led clinics.

A number of authors [9,40] contend that questionnaires should be specific for the environment in which the clinical teaching is taking place. Therefore, when exploring on-campus, student-led clinics the use of previously developed validated questionnaires, particularly those developed for in-patient or ambulatory environments, are considered unsuitable. The current paper reports on the development of a purposefully designed questionnaire to evaluate clinical teaching in an on-campus, student-led osteopathic teaching clinics at one Australian university.

## Methods

**Figure 1:**
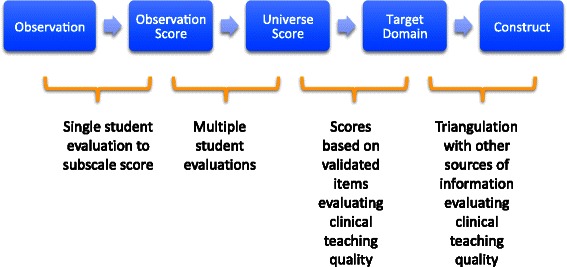
**Kane’s validity argument.**

The current study is the first in a series of studies using Kane’s validity perspective [41] to develop a fit for purpose evaluation tool, to identify clinical educators knowledge, skills and abilities by students in a student-led, on campus ambulatory clinic. The current study sought to begin developing the validity argument for the evaluation tool. Kane [41] contends that it is not possible for a measurement in itself to be ‘valid’ however it is possible to develop and mount an argument that the score itself is ‘valid’ based on multiple sources of evidence [42]. Cook [43] defines this as “…degree to which the interpretations of scores resulting from an assessment [measurement] activity are ‘well grounded or justifiable’”. Kane’s validity perspective was used as the framework for the current study and sought to provide evidence for the ‘observation’ to ‘target domain’ components of the argument (Figure 1). The study was undertaken in four phases and was approved by the Victoria University Human Research Ethics Committee.

### Phase 1 - literature review

**Figure 2:**
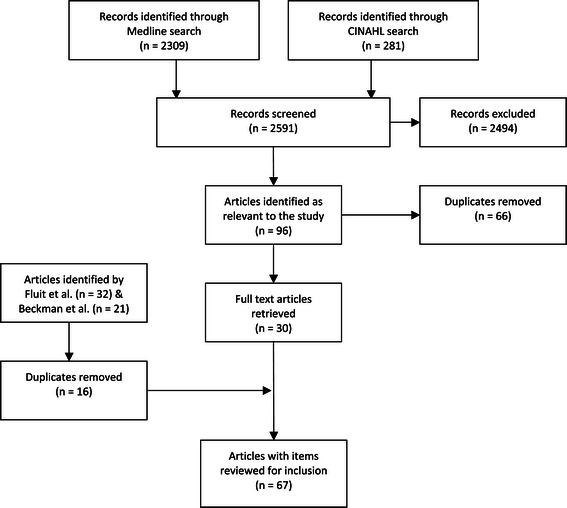
**Search strategy.**

The questionnaires identified in the systematic reviews by Fluit [35] and Beckman et al. [33] were retrieved in the first instance. To ensure the literature review for the current study was up-to-date, a further search, using the search terms outlined by Fluit [35] was undertaken from the end of the Fluit [35] review (end of March 2010) to 1^st^ January 2013. Medline and CINAHL were searched as per the Fluit [35] review and English language studies only were retrieved. Articles were retrieved where the title and/or abstract suggested the development or validation of a measure of clinical teaching quality. An overview of the search is presented in Figure 2 and Additional file 1.

Questionnaires identified from both systematic reviews, and those located during the updated search were independently reviewed by the author and an academic colleague. Items from each of the questionnaires were extracted where they were deemed to be relevant to a questionnaire evaluating clinical teaching quality in a student-led, on campus clinic. Where there was disagreement between the authors, a consensus was reached. The authors agreed on the extraction of eighty-three (83) items from twenty-three (23) questionnaires.

### Phase 2 – item review

Utilising the process employed by Roff et al. [44] to develop their clinical teaching questionnaire, 5 osteopathic clinical educators and academics, and 3 students in the VU osteopathy program responded to an invitation to review each of the 83 items. Using a 5-point Likert scale - 1 (strongly disagree) to 5 (strongly agree), the respondents were asked to rate whether the item should be included in a questionnaire about osteopathic student-led, on-campus teaching clinics. Once the respondents had completed their review of the items, the author (BV) collated the responses. Items where 6 or more of the respondents provided a rating of 4 or 5 on the Likert Scale were retained. This provided a list of 56 items and 2 global rating items. An additional global item was suggested by one of the academics and was subsequently included in the Phase 3 questionnaire. This third global rating is similar to that used in the patient satisfaction literature where a patient would recommend the particular facility to another person [45]. On the draft clinical teaching quality questionnaire, the student is asked whether they would recommend the clinical educator to other students and provides the contrast to the first global rating asking the student whether they would work with the clinical educator in the future.

### Phase 3 - questionnaire pilot testing

The draft clinical teaching quality questionnaire was distributed as a paper-based questionnaire to all students in year 4 and 5 of the VU osteopathy program. Each student was asked to complete the questionnaire and rate two of their clinical educators who had supervised them over semester 1, 2013 (March 2013 - May 2013). The students were asked to name the clinical educator on the survey, however they were not required to identify themselves. Each item in the draft clinical teaching quality questionnaire was rated on a 5-point Likert scale: 5 being ‘strongly agree’ and 1 being ‘strongly disagree’. Previous research suggests between 4 and 7 ordinal responses is best [46], as it may allow for neutral responses, and a sufficient range of responses to each item [47]. Student responses to the questionnaire were made available to each of the clinical educators who received a rating(s) from any student. The results were used for feedback purposes only and were not used as a basis for employment decisions or reward.

### Phase 4 - data analysis

Data from each completed questionnaire were entered into Microsoft Excel for quantitative analysis. Many of the questionnaires used to evaluate the quality of clinical teaching that have been published in the literature, and where the items for the current questionnaire were extracted from, have used a principal components analysis (PCA). Numerous authors have discussed the pros and cons of using a PCA [48,49], and it is now accepted that it is more appropriate to use an exploratory factor analysis (EFA) over a PCA [48], particularly where confirmatory factor analysis is to be used in the future [49,50].

There is also a move away from the use of Pearson correlations with EFAs to polychoric correlations. The polychoric correlation is more appropriate for ordinal data as Pearson correlations assume that the data has been measured on an interval scale [50-52]. Determining the number of factors to extract is traditionally based on the K1 criteria (eigenvalues greater than 1) and visual inspection of the Scree plot are both problematic - K1 has a tendency to overestimate the number of factors to be extracted [53]. Authors are now reporting the use of other methods to determine the number of factors to extract including PA [54], Velicer’s MAP [55,56], VSS [57], OC and AF [58], although such techniques have existed in the literature for many years. Of these methods, the most accurate is PA using the polychoric matrix [59]. Readers are directed towards other authors for further discussion of the factor extraction methods [48,52,59].

The exploratory factor analysis (EFA) was conducted with R [60] using the *psych* [61], *GPArotation* [62], *polycor* [63] and *nFactors* [64] packages. Data were screened and determined to be non-normally distributed. Initially a polychoric correlation matrix was generated. Polychoric correlations are more appropriate than Pearson correlations for ordinal data as they are based on the concept that the ordinal categories are bivariate normal [59].

Multiple methods were employed to determine the number of factors to extract. Parallel analysis (PA) [54], mean average partial (MAP) [55,56], eigenvalues, Very Simple Structure (VSS) [57], acceleration factor (AF) [58] and optimal coordinate (OC) [58] were all undertaken, each using the previously generated polychoric correlations. Both PA and OC have been reported to provide similar results, albeit using Pearson correlations [53].

An EFA was performed on the polychoric correlation [52] using the ordinary least squares (OLS) extraction method [48]. The questionnaire data were not normally distributed and ordinal in nature therefore the OLS extraction method should be used with the polychoric matrix [48]. Further, two rotation criteria were employed as the choice of criteria may produce different results [65,66]. Orthogonal rotations (i.e. Varimax) are commonly employed and assume that there is no correlation between the factors extracted [48]. Conversely, where the factors are expected to correlate (as in the present study) an oblique rotation is more appropriate [48]. The Geomin and Oblimin rotations were selected in the present study to reduce the cross-loadings between factors [48], and anticipating that each factor would correlate with the others. Items were retained if they loaded greater than 0.45 on a factor [67,68], had a communality of greater than 0.6 [69], and demonstrated a cross-loading of less than 0.32 [68]. After an item was removed, the EFA was conducted again (iteration) [68]. The Kaiser-Myer-Olkin (KMO) statistic and Bartlett’s test of sphericity were also calculated to determine factorability of the data.

Once the factor analysis was completed, descriptive statistics were generated for each retained item, and internal consistency of each of the factors was calculated using ordinal reliability alpha [70]. Ordinal reliability alpha is the most appropriate internal consistency statistic for ordinal data as it uses the polychoric correlation rather than the Pearson correlation [70,71]. Descriptive statistics were also generated for the three global ratings items. Descriptive statistics for the total questionnaire score and internal consistency for the whole questionnaire were not calculated as dimensionality of the questionnaire was not assessed. Dimensionality of the questionnaire will be the subject of future research.

## Results

**Figure 3:**
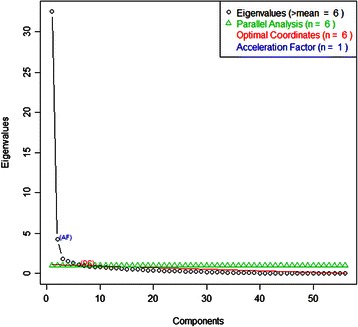
**Number of factors to extract (part 1).**

**Figure 4:**
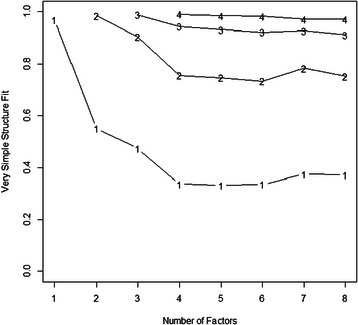
**Number of factors (part 2).** VSS plots the goodness of fit statistic as a function of the number of factors to extract. The number of factors to extract is demonstrated when the goodness of fit value no longer changes. In the above graph, the VSS fit statistic does not change when extracting four factors.

One hundred and seventy two ratings of all 27 clinical educators employed at the time of study were received. All clinical educators received more than one rating. Data were incomplete on one questionnaire and was subsequently removed from the analysis; 171 questionnaires were analysed. The results of the PA, MAP, VSS, eigenvalue, OC and AF are presented in Figures 3 and 4. The MAP suggested extracting two factors and the VSS suggested extracting four. OLS factor analyses were conducted extracting between 3–6 factors in order to identify an appropriate structure, consistent with recommendations from previous authors [59]. Eight analyses were conducted; four using the Geomin rotation and four using the Oblimin rotation. Extracting four and five factors using the Oblimin rotation provided the most appropriate solutions for (Table 1).

**Table 1:** **Factor solution choice**

Number of factors	Extraction	Rotation	Comment
3	OLS	Geomin	12 items with communalities less than 0.6, removing these items would also remove one factor
4	OLS	Geomin	9 items with communalities less than 0.6, strong cross-loadings
5	OLS	Geomin	One item on factor 5, and strong cross-loadings between factors 1 and 4
6	OLS	Geomin	Single items on factors 5 and 6, factor 6 item cross-loads with factor 1
3	OLS	Oblimin	Failed to converge
4	OLS	Oblimin	More than 5 items on each factor, minimal number of cross-loading items, logical items grouping for each factor
5	OLS	Oblimin	Logical item grouping for each factor
6	OLS	Oblimin	Failed to converge

OLS – ordinary least squares.

### Four factor solution

The 4-factor solution initially demonstrated minimal cross-loadings and slightly lower communalities than the 5 factor solution. KMO was 0.6 and Bartlett’s test was p < 0.01 (χ^2^ = 3468.40) indicating a minimally-acceptable level of factorability. Twenty-four iterations were performed; the final solution collapsed into a 3-factor structure containing 19 items explaining 77% of the variance (Table 2). The alpha scores were high (0.93 or greater) and the correlations between the three factors were 0.57 or higher. Retained items loaded greater than 0.54 on a factor and had communalities (h^2^) of greater than 0.63.

**Table 2:** **3 factor solution**

Item	F1	F2	F3	h ^ 2 ^
Treated me with respect	1.01			0.96
Maintained a positive attitude towards me	1.01			0.91
Fostered an environment of respect in which I felt comfortable participating	0.85			0.95
Showed genuine concern for my professional well-being	0.76			0.75
Established a good learning environment (approachable, focused, nonthreatening, professional and enthusiastic	0.76			0.87
Had reasonable expectations of students	0.75			0.69
Demonstrated humanistic attitudes in relating to patients (integrity, compassion and respect)	0.7			0.66
Has good communication skills	0.63			0.68
Gave me the opportunity to offer opinions on patient problems or treatment	0.54			0.69
Gave timely feedback to me		0.92		0.79
Gave me regular, useful feedback about my knowledge and performance		0.88		0.87
Offered me suggestions for improvement when required		0.87		0.86
Identified my strengths		0.66		0.63
Explained to me why I was correct or incorrect		0.64		0.77
Encouraged me to think			0.81	0.78
Asked questions that promote learning (clarifies, probes, reflective questions etc.)			0.8	0.74
Encouraged questions and active participation			0.79	0.87
Stimulates me to learn independently			0.79	0.73
Asked questions to enhance my learning			0.68	0.72
Variance explained	35%	22%	20%	
Internal consistency	0.96	0.93	0.94	

The descriptive statistics for each of the items in the 3-factor solution are presented in Table 3.

**Table 3:** **Descriptive statistics for 3-factor solution**

Item descriptives	Mean	SD	Median	Min	Max	Range
Treated me with respect	4.4	0.97	5	1	5	4
Maintained a positive attitude towards me	4.37	0.92	5	1	5	4
Fostered an environment of respect in which I felt comfortable participating	4.16	1.1	5	1	5	4
Showed genuine concern for my professional well-being	3.24	0.89	3	1	4	3
Established a good learning environment (approachable, focused, nonthreatening, professional and enthusiastic	4.19	1.07	5	1	5	4
Had reasonable expectations of students	3.23	0.87	3	1	4	3
Demonstrated humanistic attitudes in relating to patients (integrity, compassion and respect)	4.41	0.81	5	1	5	4
Has good communication skills	4.19	1.02	5	1	5	4
Gave me the opportunity to offer opinions on patient problems or treatment	4.34	0.89	5	1	5	4
Gave timely feedback to me	3.95	1.03	4	1	5	4
Gave me regular, useful feedback about my knowledge and performance	3.79	1.03	4	1	5	4
Offered me suggestions for improvement when required	4.14	0.89	4	1	5	4
Identified my strengths	3.72	1.04	4	1	5	4
Explained to me why I was correct or incorrect	3.09	0.93	3	1	4	3
Encouraged me to think	4.24	0.86	4	1	5	4
Asked questions that promote learning (clarifies, probes, reflective questions etc.)	4.12	0.93	4	1	5	4
Encouraged questions and active participation	4.17	0.88	4	1	5	4
Stimulates me to learn independently	4.03	0.9	4	1	5	4
Asked questions to enhance my learning	4.18	0.93	4	1	5	4

### Five factor solution

The 5-factor solution initially demonstrated minimal cross-loadings and higher communalities than the 4-factor solution. KMO was 0.79 and Bartlett’s test was p < 0.001 (χ^2^ = 31046.67) indicating acceptable factorability. The 5-factor structure was maintained after 18 iterations and contained 30 items explaining 80% of the variance (Table 4). Retained items loaded greater than 0.51 on a factor with communalities (h^2^) greater than 0.67. The alpha scores for each factor were above the acceptable level of 0.70 and correlations between the factors were greater than 0.24.

**Table 4:** **5 factor solution**

Item	F1	F2	F3	F4	F5	h ^ 2 ^
Treated me with respect	0.99					0.93
Maintained a positive attitude towards me	0.97					0.90
Fostered an environment of respect in which I felt comfortable participating	0.88					0.90
Established a good learning environment (approachable, focused, nonthreatening, professional and enthusiastic)	0.85					0.88
Demonstrated humanistic attitudes in relating to patients (integrity, compassion and respect)	0.80					0.73
Was approachable for discussion	0.78					0.87
Showed genuine concern for my professional well-being	0.77					0.79
Had reasonable expectations of students	0.76					0.73
Has good communication skills	0.69					0.77
Is open to student questions and alternative approaches to patient management	0.68					0.75
Gave me the opportunity to offer opinions on patient problems or treatment	0.59					0.72
Adjusted teaching to my needs (experience, competence, interest)	0.52					0.71
Is an effective clinical teacher	0.51					0.87
Encouraged me to think		0.86				0.84
Promoted reflection on clinical practice		0.83				0.67
Emphasises a problem-solving approach rather than solutions		0.78				0.68
Asked questions that promote learning (clarifies, probes, reflective questions etc.)		0.75				0.77
Asked questions to enhance my learning		0.70				0.75
Encouraged questions and active participation		0.65				0.82
Stimulates me to learn independently		0.62				0.70
Gave timely feedback to me			0.89			0.85
Gave me regular, useful feedback about my knowledge and performance			0.77			0.86
Offered me suggestions for improvement when required			0.76			0.86
Identified areas needing improvement			0.69			0.71
Identified my strengths			0.65			0.67
Explained to me why I was correct or incorrect			0.58			0.80
Promoted keeping of medical records in a way that is thorough, legible, efficient and organised				0.96		0.92
Encouraged me to assume responsibility for patient care				0.76		0.84
Demonstrates knowledge of current medical and manual therapy literature					0.89	0.85
Demonstrated osteopathic, clinical examination and rehabilitation knowledge and skill(s)					0.57	0.74
Variance explained	39%	24%	19%	9%	8%	
Internal consistency	0.97	0.94	0.93	0.82	0.73	

The descriptive statistics for each of the items in the 5-factor solution are presented in Table 5.

**Table 5:** **Descriptive statistics for the 5-factor solution**

Item	Mean	SD	Median	Min	Max	Range
Treated me with respect	4.40	0.97	5	1	5	4
Maintained a positive attitude towards me	4.37	0.92	5	1	5	4
Fostered an environment of respect in which I felt comfortable participating	4.16	1.10	5	1	5	4
Established a good learning environment (approachable, focused, nonthreatening, professional and enthusiastic)	4.19	1.07	5	1	5	4
Demonstrated humanistic attitudes in relating to patients (integrity, compassion and respect)	4.41	0.81	5	1	5	4
Was approachable for discussion	4.38	0.94	5	1	5	4
Showed genuine concern for my professional well-being	3.24	0.89	3	1	4	3
Had reasonable expectations of students	3.23	0.87	3	1	4	3
Has good communication skills	4.19	1.02	5	1	5	4
Is open to student questions and alternative approaches to patient management	4.21	0.99	5	1	5	4
Gave me the opportunity to offer opinions on patient problems or treatment	4.34	0.89	5	1	5	4
Adjusted teaching to my needs (experience, competence, interest)	4.05	0.97	4	1	5	4
Is an effective clinical teacher	4.23	1.04	5	1	5	4
Encouraged me to think	4.24	0.86	4	1	5	4
Promoted reflection on clinical practice	4.19	0.85	4	1	5	4
Emphasises a problem-solving approach rather than solutions	4.11	0.93	4	1	5	4
Asked questions that promote learning (clarifies, probes, reflective questions etc.)	4.12	0.93	4	1	5	4
Asked questions to enhance my learning	4.18	0.93	4	1	5	4
Encouraged questions and active participation	4.17	0.88	4	1	5	4
Stimulates me to learn independently	4.03	0.9	4	1	5	4
Gave timely feedback to me	3.95	1.03	4	1	5	4
Gave me regular, useful feedback about my knowledge and performance	3.79	1.03	4	1	5	4
Offered me suggestions for improvement when required	4.14	0.89	4	1	5	4
Identified areas needing improvement	3.93	0.95	4	1	5	4
Identified my strengths	3.72	1.04	4	1	5	4
Explained to me why I was correct or incorrect	3.09	0.93	3	1	4	3
Promoted keeping of medical records in a way that is thorough, legible, efficient and organised	4.20	0.82	4	1	5	4
Encouraged me to assume responsibility for patient care	3.39	0.69	3.5	1	4	3
Demonstrates knowledge of current medical and manual therapy literature	4.23	0.86	4	1	5	4
Demonstrated osteopathic, clinical examination and rehabilitation knowledge and skill(s)	4.39	0.82	5	1	5	4

### Global ratings

The descriptive statistics for the three global rating items are presented in Table 6.

**Table 6:** **Descriptive statistics for the total score and global ratings**

	I would do more clinics with this Clinical Educator	Rate the overall effectiveness of this Clinical Educator as an educator/supervisor	I would recommend other students to work with this Clinical Educator
**Mean**	4.09	4.10	4.09
**SD**	1.15	0.98	1.12
**Median**	4.50	4.00	4.00
**Minimum**	1	1	1
**Maximum**	5	5	5

## Discussion

The aim of the current paper was to develop a questionnaire to evaluate the quality of clinical teaching in an osteopathic student-led, on-campus teaching clinic at one Australian university. A systematic search of the clinical teaching evaluation literature identified questionnaires from which 83 possible items for inclusion on the new questionnaire were extracted. The extraction of these items was based on their perceived applicability to a student-led, on-campus teaching clinic environment. Items were drawn from published questionnaires designed for a range of clinical teaching environments. No questionnaire assessing clinical teaching quality in student-led clinics or ambulatory, on-campus clinics was identified. As a questionnaire should be designed for the environment in which it is to be used [9,40], drawing on these previously published items is appropriate for developing the questionnaire for a student-led, on-campus clinic. By employing the same method as Roff et al. [44], osteopathy academics, clinical educators and students refined the list of 83 items to 56 items. During this process items that related to the conduct of formative and summative assessments by the clinical educators were removed as the content reviewers felt this was not a role that the students could provide constructive ratings for nor were the assessments a major role undertaken by the clinical educators. The resulting draft clinical teaching quality questionnaire was then completed by the cohort of osteopathy students who were managing patients in the student-led clinic at the time of the study.

To determine the most appropriate items and factor structure for a questionnaire to assess the quality of clinical teaching in the student-led on-campus clinic, results from the 56-item questionnaire were analysed with an EFA. Multiple methods were used to determine the number of factors to extract withthe PA, MAP, and VSS all suggesting different numbers of factors be extracted. Where the number of factors to be extracted differs between methods, Courtney and Gordon [59] suggest that multiple factors be extracted guided by the results of the PA and MAP. In the present study PA suggested extracting six factors and MAP four factors. To maximise the ability to identify an appropriate factor structure, three, four, five and six factors were extracted using both the Geomin and Oblimin oblique rotations. A number of analyses were undertaken with the most appropriate factor structures being a 4-factor and 5-factor solution. The VSS suggested extracting four factors however none of the methods suggested extracting five factors. This outcome supports the assertion of Courtney and Gordon [59] that a multiple numbers of factors should be extracted where the different methods are not in agreement.

Further analysis of the 4 and 5 factor solutions, including item removal based on multiple criteria, produced a 3-factor and 5-factor structure respectively. It is of note that the 4-factor solution collapsed to a 3-factor solution due to the removal of some of the items. The initial factorability of the 4-factor solution was minimally acceptable, and may have been an indicator as to the potential for the factor structure to collapse during the analysis. The decision was made to use the 5-factor, 30-item questionnaire as it displayed characteristics of previously validated questionnaires [27,72], and also incorporated modelling behaviours (e.g. interacting with patients and professional practice) that were not being examined by items remaining in the 3-factor structure. The 5-factor, 30-item questionnaire was called the Osteopathy Clinical Teaching Questionnaire (OCTQ). The five factors identified in the present study were labelled: Learning Environment (Factor 1), Reflective Practice (Factor 2), Feedback (Factor 3) and Patient Management (Factor 4) and Modelling (Factor 5).

### Factor 1 - learning environment

The clinical learning environment is a confluence of factors. This includes those listed previously (i.e. patient census) as well as system-based considerations such as the requirements of accrediting bodies, university requirements (i.e. graduate attributes), operational issues (i.e. physical clinic environment, clinic operating procedures), and interpersonal issues (i.e. patients, administrative staff, peers, clinical educators) that students must learn to cope with and manage. These system-based influences expose the student to issues that they will experience in the workplace through their training program or upon graduation. Managing such influences is part of becoming a capable health professional [73].

Griffith III et al. [74] indicate that the learning environment, managed and/or facilitated by the clinical educator, improves student learning more than the clinical educator imparting information. Furthermore the learning environment effects the overall judgement of the clinical teaching as rated by a student [1,27,30,31,72]. Boerboom et al. [72] noted that over 20% of the variance in the Maastricht Clinical Teaching Questionnaire scores was accounted for by the modelling, coaching and learning climate domains – factors directly related to the clinical teacher and the learning environment. In the present study, Learning Environment (Factor 1) was the strongest factor and accounted for just over a third of the variance in the data (39%). This factor also demonstrated a strong ordinal alpha value indicating that it is internally consistent, and contained items that are measuring a similar construct.

Students feel that a positive relationship with the clinical educator contributes to a favourable learning environment [75] and this is reflected in a higher score on the OCTQ. The potential for this relationship or ‘halo effect’ to influence item responses should be investigated further. Respect is also a key component of this factor. Whilst the focus of this factor was on the interaction between clinical educator and student, it also addressed the interaction between the clinical educator and patient (item 5). This interaction is an important part of the role of an effective clinical educator [10] and provides an opportunity for the educator to role model patient communication and management skills.

### Factor 2 - reflective practice

This factor addressed a range of areas including reflective skills (items 14, 15 & 17), and the use of questioning to promote learning (item 17, 18 & 19). A number of items on the OCTQ address reflective practice and reinforce the importance of the clinical educator stimulating self-directed learning. Litzelman et al. [76] have reported a strong positive correlation between clinical educators who stimulate self-directed learning and higher clinical teaching quality ratings. These authors [76] suggest that such a relationship is an indication of the clinical educators knowledge. However, items that require the student to actively participate in the clinical education process, *i.e. item 14* - *Encouraged me to think* may not be a true reflection of the quality of clinical education provided by the clinical educator. These items are potentially susceptible to differences in students willingness to engage with the clinical educator rather than differences in approaches clinical educators use to stimulate thinking [72]. Given the potential positive impact of the stimulation of self-directed learning on clinical teaching quality ratings [76], items such *7. Asked questions that promote learning (clarifies, probes, reflective questions etc.)* should be retained. Institutions could use this information to design professional development activities for their clinical educators to help them work with students to develop their reflective practice and self-directed learning skills.

### Factor 3 - feedback

Feedback to the student about their performance is a strong theme in the clinical teaching literature [15,27,77]. Timely feedback to the student was the strongest loading item on this factor. This result is consistent with the literature that suggests feedback should be provided to the student in a timely fashion [14,21,78,79]. The provision of both positive and negative feedback to the student are captured in the OCTQ. Further, item 26 - *Explained to me why I was correct or incorrect* allows the student to report their perception of the ability of the clinical educator to provide constructive feedback. Feedback provided to a student can be positive or negative, informal or continuous, and formative, or based around summative assessments such as the mini Clinical Examination (mini-CEX) [80,81]. What is not captured in this factor is the quality of the feedback provided by the clinical educator, and whether this had an impact on the future performance of the student. This is an area that could be explored in the future.

### Factor 4 - patient management

The role of the clinical educator in the student-led clinic is to oversee the student writing the clinical history, and an expectation is that the educator will promote best practice in relation to record keeping. This is captured in item 27 - *Promoted keeping of medical records in a way that is thorough, legible, efficient and organised*. This type of item is not common in clinical teaching quality questionnaires, and given the importance of case notes for practitioner communication and medicolegal reasons, it is felt that this item is a valuable addition. Developers of clinical teaching evaluation questionnaires should consider the inclusion of the same or similar item in the future. It is also noteworthy that this item loaded strongly onto the factor.

Traditionally, patient care in the early stages of the students’ clinical education is scaffolded from observation through to autonomous patient care. In the student-led clinic environment, students will often have greater patient care responsibilities compared to the hospital setting [82]. In the osteopathy program at VU, students who completed the questionnaire in the present study were already responsible for patient care, under supervision. The inclusion of item 28 - *Encouraged me to assume responsibility for patient care* is relevant for the student-led clinic environment, as lower scores for this item indicates that the student may feel that the supervisor ‘takes over’ or directs the treatment and as such, the student may perceive that their responsibility for patient care has reduced. Students need to feel as though they are supported by the clinical educator when managing a patient but they have substantial autonomy in conducting the treatment.

### Factor 5 - modelling

Modelling (Factor 5) is the clinical educator taking on the responsibility of professional role model and the demonstration of the skills and knowledge that are expected of a capable health professional. Modelling was identified by Stalmeijer et al. [27] as an important determinant of the effectiveness of the clinical educator. Students in the present study appear to value the knowledge and technical skills of the clinical educator. It is important for the clinical educator to undertake professional development and reading outside of their education role in order to inform their clinical teaching. The VU osteopathy program emphasises evidence informed practice [83,84] including it in the mission statement for the program. Therefore incorporating such an item in the OCTQ is important to ensure the clinical educators are modelling appropriate behaviours. Modelling extends to the demonstration of osteopathic examination and technique, clinical examination skills (e.g. performing a cranial nerve examination) as well as rehabilitation and advice to the patient; all parts of the typical osteopathic consultation. Demonstration of these physical and clinical skills has previously been demonstrated to be behaviours of an effective clinical educator [24,26].

### Global ratings

The global ratings provide a way for the student to rate the overall performance and quality of the clinical educator. All three global ratings demonstrated mean scores greater than 4. An issue with the use of such a rating is the possibility of a ‘ceiling effect’ [22] therefore the rating needs to be interpreted in conjunction with the individual OCTQ items. With the ‘ceiling effect’ it may be difficult to differentiate between high quality clinical educators, although this may not be of great concern given they are already achieving high ratings. Working with the results of individual items may assist the clinical educator and their supervisor/manager to develop targeted professional development activities or assist with promotion decisions.

### Psychometric properties

From a statistical viewpoint, Factors 1, 2 and 3 had high alpha values whereas Factors 4 and 5 had moderate (<0.7) alpha values. These moderate values are likely due to the fact that the factors contained two items. Whilst alpha values of 0.7 above are generally considered to be the minimum acceptable [85], these factors will be retained given the relevance of the items (items 22–26). Future studies into the OCTQ will employ modern test theory approaches such as Rasch analysis in order to strength the psychometric properties of the questionnaire.

The results of the present study provide evidence for the content and face validity of the OCTQ as the items were drawn from published clinical teaching questionnaires, and examined by both the clinical educators and students who will be using the questionnaire. A unique aspect of the OCTQ is that it contains items that are specific to osteopathic clinical education (items 21, 22 and 24) and this has not been reported in the literature previously. The factors generated in the present study are generally consistent with the clinical education literature and therefore, the questionnaire could be generalisable to other student-lead, on-campus clinics.

### Limitations

Larger student populations and multiple institutions should be used in future studies to improve the generalisability of the questionnaire. A limitation of the present study was that each student only rated two clinical educators. There is the potential for bias to occur in that students may have rated those educators they wished to rate based on a positive or negative perception leading to ratings at the extremes of the scale options. Further research is required to confirm the factor structure of the questionnaire, establish the test-retest reliability, undertake a generalisability analysis to determine the number of ratings to generate a reliable result as well as examining the concurrent validity.

## Conclusions

This study has developed a questionnaire - the Osteopathy Clinical Teaching Questionnaire - to assess the quality of clinical education in a student-led teaching clinic in a pre-registration osteopathic teaching program at one Australian university. The items were identified in the literature and then tested with students in the clinical education component of the program. The OCTQ contains 30 items, and 3 global items, which address a range of behaviours and roles that students perceive to be important for an osteopathic clinical educator. The evidence-informed approach to the EFA employed in the present study helps to strengthen the construct validity of the questionnaire. This paper provides evidence for the ‘observation’ through to ‘target’ domain components of Kane’s perspective on validity. Further evidence will be sought to provide a justification of the validity of the scores derived from the OCTQ and the questionnaire will now be the subject of further investigation to establish its psychometric properties and generalisability - these will be reported on in subsequent papers. Questionnaires like the OCTQ have the potential to improve the clinical learning experience for the student and the ensuing positive impact on patient care.

## Additional file

Additional file 1:
**Systematic literature search.**
