# Supplementary material for: Developing a clinical teaching quality questionnaire for use in a university osteopathic pre-registration teaching program
Source: BMC Med Educ. 2015 Apr 8;15:70. doi: 10.1186/s12909-015-0358-6 (PMC4404120; doi:10.1186/s12909-015-0358-6)
Supplement: Additional file 1: — Systematic literature search. [file 12909_2015_358_MOESM1_ESM.pdf]

	Medline	CINAHL
**Search term**	**Articles found**	**Articles found**
Clinical teacher NOT nursing	Butani et al. [1]<br>Boerboom et al. [2]<br>Arah et al. [3]<br>Boerebach et al. [4]<br>Makama & Ameh [5]<br>Al-Qahtani [6]<br>Bell et al. [7]<br>Butvidas [8]<br>Boerboom et al. [9]<br>Stenfors-Hays et al. [10]<br>Schwartz [11]<br>Bannister et al. [12]	Al Haqwi et al. [13]<br>Arah et al. [3]<br>Schwartz [11]
Medical teacher NOT nursing AND instrument	Archer et al. [14]<br>Riquelme [15]<br>Fluit et al. [16]<br>Boerboom [2]<br>Kamran et al. [17]<br>Nation et al. [18]<br>Boor et al. [19]<br>Boerboom et al. [9]	
Medical teacher NOT nursing AND validity	Archer et al. [14]<br>van Es et al. [20]<br>Fluit et al. [16]<br>Boerboom et al. [2]<br>Yu et al. [21]<br>Nation et al. [18]<br>Boerboom et al. [9]	
Clinical teaching NOT nursing AND instrument	Schonrock-Adema et al. [22]<br>Archer et al. [14]<br>Fluit et al. [16]<br>Egbe & Baker [23]<br>Boerboom et al. [2]<br>Nation et al. [18]<br>Boerboom et al. [9]<br>Zibrowski et al. [24]<br>Stalmeijer et al. [25]	Conigliaro & Stratton [26]<br>Zibrowski et al. [24]
Clinical teaching NOT nursing AND evaluation AND instrument	Schonrock-Adema et al. [22]<br>Archer et al. [14]<br>Fluit et al. [16]<br>Egbe & Baker [23]<br>Boerboom et al. [2]<br>Nation et al. [18]<br>Boerboom et al. [9]<br>Zibrowski et al. [24]<br>Stalmeijer et al. [25]	Conigliaro & Stratton [26]<br>Zibrowski et al. [24]

Medical education AND clinical teacher	Arah et al. [3]<br>Makama & Ameh [5]<br>Schwartz [11]	Al Haqwi et al. [13]<br>Arah et al. [3]
Medical education AND effectiveness AND instrument	Schonrock-Adema et al. [22]<br>Archer et al. [14]<br>Zibrowski et al. [24]	XiaoJing [27]<br>Zibrowski et al. [24]
Medical education AND teaching AND instrument AND evaluation	Archer et al. [14]<br>Fluit et al. [16]<br>Egbe & Baker [23]<br>Arah et al. [3]<br>Nation et al. [18]<br>Zibrowski et al. [24]<br>Stalmeijer et al. [25]	Conigliaro & Stratton [26]<br>XiaoJing [27]<br>Zibrowski et al. [24]
Medical education AND teaching AND validity AND instrument	Schonrock-Adema et al. [22]<br>Archer et al. [14]<br>Fluit et al. [16]<br>Egbe & Baker [23]<br>Arah et al. [3]<br>Zibrowski et al. [24]<br>Stalmeijer et al. [25]	Conigliaro & Stratton [26]<br>XiaoJing [27]
Instrument AND clinical teacher	Bergjan & Hertel [28]<br>Henriksen et al. [29]<br>Bos et al. [30]<br>Boerboom et al. [2]<br>Boerboom et al. [9]<br>Johansson et al. [31]	Johansson et al. [31]<br>Henriksen et al. [29]<br>Bos et al. [30]
Instrument AND validity AND clinical teacher	Henriksen et al. [29]<br>Boerboom et al. [2]<br>Boerboom et al. [9]<br>Johansson et al. [31]	Johansson et al. [31]<br>Henriksen et al. [29]

Additional File 1. Articles identified and selected from the systematic search of the literature from 1st March 2010 to 1st January 2013.

REFERENCES

1. Butani L, Paterniti DA, Tancredi DJ, Li S-TT: **Attributes of residents as teachers and role models-a mixed methods study of stakeholders.** *Med Teach* 2012, **35:**e1052-e1059.
2. Boerboom TB, Mainhard T, Dolmans DH, Scherpbier AJ, Van Beukelen P, Jaarsma AC: **Evaluating clinical teachers with the Maastricht clinical teaching questionnaire: How much'teacher'is in student ratings?** *Med Teach* 2012, **34:**320-326.
3. Arah OA, Heineman MJ, Lombarts KM: **Factors influencing residents' evaluations of clinical faculty member teaching qualities and role model status.** *Med Educ* 2012, **46:**381-389.
4. Boerebach BC, Lombarts KM, Keijzer C, Heineman MJ, Arah OA: **The teacher, the physician and the person: how faculty's teaching performance influences their role modelling.** *PLoS ONE* 2012, **7:**e32089.
5. Makama J, Ameh E: **Quality of teaching provided by surgical residents: an evaluation of the perception of medical students.** *Nigerian journal of medicine: journal of the National Association of Resident Doctors of Nigeria* 2010, **20:**341-344.
6. Al-Qahtani MF: **What constitutes a good clinical teacher?** *The Journal Of The Egyptian Public Health Association* 2011, **86:**27-32.
7. Bell MA, Wales PS, Torbeck LJ, Kunzer JM, Thurston VC, Brokaw JJ: **Do personality differences between teachers and learners impact students' evaluations of a surgery clerkship?** *J Surg Educ* 2011, **68:**190-193.
8. Butvidas LD, Anderson CI, Balogh D, Basson MD: **Disparities between resident and attending surgeon perceptions of intraoperative teaching.** *The American Journal of Surgery* 2011, **201:**385-389.
9. Boerboom T, Dolmans D, Jaarsma A, Muijtjens A, Van Beukelen P, Scherpbier A: **Exploring the validity and reliability of a questionnaire for evaluating veterinary clinical teachers' supervisory skills during clinical rotations.** *Med Teach* 2011, **33:**e84-e91.
10. Stenfors-Hayes T, Hult H, Dahlgren LO: **What does it mean to be a good teacher and clinical supervisor in medical education?** *Adv Health Sci Educ* 2011, **16:**197-210.
11. Schwartz AJ: **Resident/fellow evaluation of clinical teaching: an essential ingredient of effective teacher development and educational planning.** *Anesthesiology* 2010, **113:**516-517.
12. Bannister SL, Raszka WV, Maloney CG: **What makes a great clinical teacher in pediatrics? Lessons learned from the literature.** *Pediatrics* 2010, **125:**863-865.
13. AlHaqwi AI, van der Molen HT, Schmidt H, Magzoub M: **Determinants of effective clinical learning: a student and teacher perspective in Saudi Arabia.** *Education for Health* 2010, **23:**369.
14. Archer J, Swanwick T, Smith D, O'Keeffe C, Cater N: **Developing a multisource feedback tool for postgraduate medical educational supervisors.** *Med Teach* 2013, **35:**145-154.
15. Riquelme A, Padilla O, Herrera C, Olivos T, Román JA, Sarfatis A, Solís N, Pizarro M, Torres P, Roff S: **Development of ACLEEM questionnaire, an instrument measuring residents' educational environment in postgraduate ambulatory setting.** *Med Teach* 2013, **35:**e861-e866.
16. Fluit C, Bolhuis S, Grol R, Ham M, Feskens R, Laan R, Wensing M: **Evaluation and feedback for effective clinical teaching in postgraduate medical education: Validation of an assessment instrument incorporating the CanMEDS roles.** *Med Teach* 2012, **34:**893-901.
17. Kamran A, Zibaei M, Mirkaimi K, Shahnazi H: **Designing and evaluation of the teaching quality assessment form from the point of view of the Lorestan University of Medical Sciences students-2010.** *Journal of education and health promotion* 2012, **1.**

18. Nation JG, Carmichael E, Fidler H, Violato C: **The development of an instrument to assess clinical teaching with linkage to CanMEDS roles: a psychometric analysis.** *Med Teach* 2011, **33:**e290-e296.
19. Boor K, Van Der Vleuten C, Teunissen P, Scherpbier A, Scheele F: **Development and analysis of D-RECT, an instrument measuring residents' learning climate.** *Med Teach* 2011, **33:**820-827.
20. van Es JM, Schrijver CJ, Oberink RH, Visser MR: **Two-dimensional structure of the MAAS-Global rating list for consultation skills of doctors.** *Med Teach* 2012, **34:**e794-e799.
21. Yu T-C, Wheeler BR, Hill AG: **Clinical supervisor evaluations during general surgery clerkships.** *Med Teach* 2011, **33:**e479-e484.
22. Schönrock-Adema J, Boendermaker PM, Remmelts P: **Opportunities for the CTEI: disentangling frequency and quality in evaluating teaching behaviours.** *Perspectives on medical education* 2012, **1:**172-179.
23. Egbe M, Baker P: **Development of a multisource feedback instrument for clinical supervisors in postgraduate medical training.** *Clin Med* 2012, **12:**239-243.
24. Zibrowski EM, Myers K, Norman G, Goldszmidt MA: **Relying on others' reliability: challenges in clinical teaching assessment.** *Teach Learn Med* 2011, **23:**21-27.
25. Stalmeijer RE, Dolmans D, Wolfhagen H, Muijtjens AM, Scherpbier A: **The Maastricht Clinical Teaching Questionnaire (MTCQ) as a valid and reliable instrument for the evaluation of clinical teachers.** *Acad Med* 2010, **85:**1732-1738.
26. Conigliaro RL, Stratton TD: **Assessing the quality of clinical teaching: a preliminary study.** *Med Educ* 2010, **44:**379-386.
27. XiaoJing H, Zhu D, Zheng M: **Clinical Nursing Faculty Competence Inventory–development and psychometric testing.** *J Adv Nurs* 2011, **67:**1109-1117.
28. Bergjan M, Hertel F: **Evaluating students' perception of their clinical placements—Testing the clinical learning environment and supervision and nurse teacher scale (CLES+ T scale) in Germany.** *Nurse Educ Today* 2013, **33:**1393-1398.
29. Henriksen N, Normann HK, Skaalvik MW: **Development and testing of the Norwegian version of the Clinical Learning Environment, Supervision and Nurse Teacher (CLES+ T) evaluation scale.** *Int J Nurs Educ Scholarsh* 2012, **9**.
30. Bos E, Alinaghizadeh H, Saarikoski M, Kaila P: **Validating the 'clinical learning environment, supervision and nurse teacher'CLES+ T instrument in primary healthcare settings using confirmatory factor analysis.** *J Clin Nurs* 2012, **21:**1785-1788.
31. Johansson UB, Kaila P, Ahlner‐Elmqvist M, Leksell J, Isoaho H, Saarikoski M: **Clinical learning environment, supervision and nurse teacher evaluation scale: psychometric evaluation of the Swedish version.** *J Adv Nurs* 2010, **66:**2085-2093.
